# Narcissism and the perception of failure – evidence from the error-related negativity and the error positivity

**DOI:** 10.1017/pen.2022.7

**Published:** 2023-02-09

**Authors:** Markus Mück, André Mattes, Elisa Porth, Jutta Stahl

**Affiliations:** University of Cologne, Cologne, Germany

**Keywords:** Error processing, Event-related potentials (ERP), Narcissism

## Abstract

The literature on narcissism suggests two contradictory ways how highly narcissistic individuals deal with their failures: They might avoid consciously recognising their failures to protect their ego or they might vigilantly turn towards their failures to process cues that are important for maintaining their grandiosity. We tried to dissolve these contradictory positions by studying event-related potential components of error processing and their variations with narcissism. With a speeded go/no-go task, we examined how the error-related negativity (Ne; reflecting an early, automatic processing stage) and the error positivity (Pe; associated with conscious error detection) vary with Admiration and Rivalry, two narcissism dimensions, under ego-threatening conditions. Using multilevel models, we showed that participants with high Rivalry displayed higher Ne amplitudes suggesting a heightened trait of defensive reactivity. We did not find variations of either narcissism dimension with the Pe, which would have pointed to weaker error awareness. Thus, our results only supported the second position: a heightened vigilance to errors in narcissism at early, rather automatic processing stages.

## Introduction

Humans strive to see themselves in a positive light (Pincus, Cain, & Wright, 2014). To pursue experiences of Admiration and self-enhancement reflects a basic psychological need inherent in all of us (Grawe, 2004). However, in narcissism, this need gains exceptional importance, rigidly determines mental functioning (affecting motivation, emotion, cognition, behaviour, and perception) and alienates the person from other fundamental psychological needs that are important for well-being and psychological functioning (Grawe, 2004; Sachse, 2013). To date, we do not fully understand how this excessive need to enhance and protect one’s grandiosity impacts the perception of failure – whether these failures relate to everyday missteps, like realizing a spelling mistake in a text message, or more dreadful experiences, like crashing one’s entrepreneurial endeavour. Do highly narcissistic people steer their attention away from such failures and inhibit further processing as failures endanger their mental representation of grandiosity? Or do they turn towards their failures and exhaustively process them as they provide essential cues for self-enhancement and ego protection? The concepts of cognitive avoidance and vigilance capture these ostensibly diametrical coping dispositions (Hock, Krohne, & Kaiser, 1996). We aimed at investigating whether there is electrophysiological and behavioural evidence for narcissism-related variations in cognitive avoidance of *and* vigilance to self-caused failures.

### 1.1 Narcissism and information processing

#### 1.1.1 Cognitive Avoidance vs. Vigilance

Several empirical studies suggest that highly narcissistic individuals particularly use cognitive avoidance to cope with failures. For example, highly narcissistic people self-aggrandise by more strongly attributing failures to external causes – and success to their own abilities (Kernis & Sun, 1994; Rhodewalt, Tragakis, & Finnerty, 2006; Stucke, 2003). They assess their current performance and predict their future performance mainly based on their inflated ability estimates and less on their actual past performance (Campbell, Goodie, & Foster, 2004). With this overestimation of their abilities, highly narcissistic people manage to sustain their grandiosity – even though overconfidence and risk-taking can worsen their actual performance (Campbell et al., 2004). They also avoid using the first-person perspective when recalling shameful events and rather revert to the third-person perspective (Marchlewska & Cichocka, 2017). Not least, highly narcissistic entrepreneurs seem to be less capable of learning from and interpreting their business failures (Liu, Li, Hao, Zhang, 2019).

However, instead of cognitively avoiding their failures, highly narcissistic individuals might show vigilance towards them. It was shown that highly narcissistic people reacted to everyday failures with lower state self-esteem levels (than low-narcissistic people; Zeigler-Hill, Myers, & Clark, 2010). So, instead of leaving them unaffected, failures might disturb highly narcissistic individuals even more. Grapsas, Brummelman, Back, and Denissen (2020) even incorporated vigilance as a central element in their process model of narcissistic status pursuit. Following this model, errors might represent important cues highly narcissistic individuals vigilantly turn to in order to manoeuvre through ego-threatening situations.

Thus, highly narcissistic individuals might either reduce error processing to avoid conscious awareness of imperfection or enhance error processing to better regulate their behaviour in the pursuit of grandiosity. Which of these seemingly contradicting coping strategies highly narcissistic individuals employ may depend, as we assume, on different *narcissism facets* and the *temporal dynamics* of information processing.

#### 1.1.2 Variations in information processing with different narcissism facets

Based on previous non-error-specific findings, we assumed that vigilance to and avoidance of one’s errors could vary distinctively with different narcissism facets. For example, it was demonstrated that the two narcissism facets *grandiosity* and *vulnerability* are related to different attention biases: away from (grandiosity) and towards (vulnerability) negative trait adjectives (Krusemark et al. 2015). These variations in attentional selection could point to differences in processing aversive errors.

Also, the two narcissism facets *Admiration* and *Rivalry* (Back et al., 2013) might vary differently with error processing. For the two facets, different self-esteem stability was observed: whereas Admiration was related to stable self-esteem, Rivalry was associated with self-esteem fragility (Geukes et al., 2017). Assuming that self-esteem stability varies with the processing of ego-threatening errors, one can assume that these facets are also associated with different error processing activity. The Narcissistic Admiration and Rivalry Concept (NARC; Back et al., 2013) regards these facets as two ways how narcissistic individuals can maintain their grandiosity. They can either pursue other people’s Admiration by seeking out opportunities to present one’s uniqueness, indulging in grandiose fantasies, and behaving charmingly towards others (Admiration). Or they can restlessly protect their grandiosity against ego threats (from other people) by striving for superiority, devaluating other people, and acting aggressively towards them (Rivalry). Through positive feedback loops between these strategies and their outcomes (Admiration: praise, social status, success, etc.; Rivalry: unpopularity, criticism, rejection, etc.) both strategies rigidify over time (Back et al., 2013). In contrast to earlier conceptualisations, the NARC separates agentic (Admiration) and antagonistic (Rivalry) aspects of narcissism. This distinction is in line with more recent models such as the Trifurcated Model (TM; Miller et al., 2016) and the Narcissism Spectrum Model (NSM; Krizan & Herlache, 2018), which also capture agentic traits (with the factors *Agentic Extraversion* [TM] and *Grandiosity* [NSM]) and antagonistic traits (with the factors *Antagonism* [TM] and *Entitlement* [NSM]). We used the NARC as a theoretical framework to explore how the narcissistic need to maintain one’s grandiosity varies with error processing. Yet, when one wants to integrate the current data into a broader research context, one could assume similar results for corresponding agentic and antagonistic factors of other narcissism models.

#### 1.1.3 Variations in temporal dynamics

We further assumed that vigilance to and avoidance of errors may vary with the temporal dynamics of information processing. Mental operations unfold and vanish in a range of milliseconds as indicated by electrophysiological markers (Luck, 2014), and identifying these temporal dynamics in neural processing poses a critical aspect in social cognitive and affective neuroscience (Amodio, Bartholow, & Ito, 2014). A study by Horvath and Morf (2009; replicated by Hardaker, Sedikides, & Tsakanikos, 2019), emphasised this point: Analysing response time (RT) data, the authors demonstrated (using a priming task followed by a lexical decision task) that at early stages of information processing, highly narcissistic individuals were hypersensitive to ego-threatening prime words; however, at later stages, they automatically and successfully prevented experiences of worthlessness from surfacing. The Mask Model of narcissism suggests that narcissistic grandiosity serves as a defensive response that masks feelings of worthlessness and inferiority (Akhtar & Thomson, 1982; Kernberg, 1975; Kohut, 1977; Morf & Rhodewalt, 2001; Miller, Lynam, Hyatt, & Campbell, 2017). Thus, conscious avoidance of one’s errors (at later error processing stages) might shield one’s grandiosity against the emergence of vulnerable states caused by one’s vigilance towards errors (at early error processing stages). In light of these considerations, it appears inevitable to apply research methods that respect the temporal dynamics in information processing to better comprehend how highly narcissistic individuals respond to their failures.

So far, most studies that are informative on the question of how highly narcissistic individuals deal with their failures mainly examined self-report data (Campbell et al., 2004; Kernis & Sun, 1994; Liu et al., 2019; Rhodewalt et al., 2006; Stucke, 2003; Zeigler-Hill et al. (2010);) and RT data (Horvath & Morf, 2009; Krusemark et al. 2015; Hardaker et al., 2019). To our knowledge, no study has investigated neural responses to errors as more direct indicators of how highly narcissistic individuals cope with failures. To fill this gap, we studied error-specific components of the event-related potential (ERP) to deepen our understanding of error processing in narcissism. The ERP technique appears advantageous for this research question for several reasons: It differentiates perceptual processes in a millisecond range (Amodio et al., 2014; Luck, 2014), provides implicit data circumventing the self-enhancing bias in narcissism (Cascio, Konrath, & Falk, 2015; Di Sarno, Di Pierro, & Madeddu, 2018; Raskin, Novacek, & Hogan, 1991;), and respects the contiguity between narcissism-relevant events and narcissism-characteristic responses (Hardaker et al., 2019).

#### 1.1.4 The error negativity

The first ERP component we examined was the error negativity (*N*_e_; Falkenstein, Hohnsbein, Hoormann, & Blanke, 1991; Gehring, Goss, Coles, Meyer, & Donchin, 1993). The *N*_e_ appears as a negative deflection in the EEG after an error in a variety of tasks (Gehring, Goss, Coles, Meyer, & Donchin, 2018), peaks from 50 to 100 ms after an error, and mainly emerges from the anterior cingulate cortex (ACC; Dehaene, Posner, & Tucker, 1994; Debener et al., 2005; Luu, Tucker, & Makeig, 2004; Holroyd, Dien, & Coles, 1998; Miltner et al., 2003; Trujillo & Allen, 2007). After correct responses, a similar, slightly weaker component occurs: the correct response negativity (*N*_c_), which resembles the *N*_e_ regarding its time course and topography (Vidal, Hasbroucq, Grapperon, & Bonnet, 2000) and possibly reflects the same process as the *N*_e_ (Hoffmann & Falkenstein, 2010). The *N*_e_ may signal the need for an increase in cognitive control (see the *Conflict Monitoring Theory* [Botvinick, Braver, Barch, Carter, & Cohen, 2001] and the *Reinforcement Learning Theory* [Holroyd and Coles, 2002]). In this view, the ACC signals to other brain regions that performance adjustments are necessary to achieve one’s action goals (Holroyd & Yeung, 2012; Ridderinkhof, Ullsperger, Crone, & Nieuwenhuis, 2004). Also, the *N*_e_ seems to be associated with affective-motivational aspects of error processing. Higher *N*_e_ amplitudes were demonstrated for higher self-reported negative affect (Hajcak, McDonald, & Simons, 2004; Luu, Collins, & Tucker, 2000), higher scores of worry and general anxiety (Hajcak, McDonald, & Simons, 2003), obsessive-compulsive disorders (OCD; e.g. Endrass et al., 2010; Gehring, Himle, & Nisenson, 2000; Weinberg, Dieterich, & Riesel, 2015), subclinical symptoms of OCD (Grundler, Cavanagh, Figueroa, Frank, & Allen, 2009; Hajcak & Simons, 2002), social anxiety disorders (Endrass, Riesel, Kathmann, & Buhlmann, 2014), and generalized anxiety disorders (Weinberg, Riesel, & Hajcak, 2012; Xiao et al., 2011). Especially, *anxious apprehension* (worry) might account for these findings as high worries stress one’s cognitive capacities, and the resulting cognitive deficits may be coped with increased error monitoring (Moser et al., 2013). Other studies, focusing on motivational aspects of error processing showed that the *N*_e_ varied with the monetary value of errors (Potts, 2011), with the external evaluation of one’s performance (Hajcak, Moser, Yeung, & Simons, 2005), with the error context (competitive vs. cooperative context; García Alanis, Baker, Peper, & Chavanon, 2019), and with aversive sounds contingently following errors (Saunders, Milyavskaya, & Inzlicht, 2015). Furthermore, the *N*_e_ varied with individual differences inherently related to a different error processing motivation like a pronounced behavioural inhibition system (Amodio et al., 2008) and perfectionism (Mattes, Mück, Stahl, 2022a; Stahl, Acharki, Kresimon, Völler, & Gibbons, 2015). Weinberg and colleagues (2012) integrated this diverse literature. They emphasised that errors threaten an organism and its goals and assumed that the *N*_e_ represents the first evaluation of this threat’s significance. The process reflected in the *N*_e_ might elicit a variety of cognitive and affective-motivational processes, altogether constituting a defensive response to an endogenous threat (Weinberg et al., 2015). According to this account, the *N*_e_ reflects a neurobehavioural trait, a stable tendency to mobilise defensive systems, which the authors termed *trait defensive reactivity* (Weinberg et al., 2012).

#### 1.1.5 The error positivity

The *N*_e_ is followed by a positive deflection in the ERP, the error positivity (*P*_e_; Falkenstein, Hohnsbein, & Hoormann, 1994; Falkenstein, Hoormann, Christ, & Hohnsbein, 2000). The *P*_e_ shows a more diffuse, centroparietal distribution (Nieuwenhuis, Ridderinkhof, Blom, & Kok, 2001; Vocat, Pourtois, & Vuilleumier, 2008). At least partially, the *N*_e_ and the *P*_e_ seem to reflect functionally dissociable error monitoring systems (Di Gregorio, Maier, & Steinhauser, 2018; Mattes, Porth, & Stahl, 2022b). While the *N*_e_ seems to reflect one’s trait defensive reactivity (Weinberg et al., 2012), the *P*_e_ is thought to reflect processes that are related to error awareness (Nieuwenhuis et al., 2001). The *Error-Awareness Hypothesis* of the *P*_e_ (Overbeek, Nieuwenhuis, & Ridderinkhof, 2005) builds on findings that the *P*_e_ (but not the *N*_e_) was higher for consciously perceived than unperceived errors (Nieuwenhuis et al., 2001) and that hypnosis, which weakens error awareness, only reduces the *P*_e_ but not the *N*_e_ (Kaiser, Barker, Haenschel, Baldeweg, & Gruzelier, 1997). Not least, Murphy, Robertson, Allen, Hester, and O’Connell (2012) reported that the timing of error awareness, indicated by the latency of an error signalling response, correlated with the *P*_e_ peak latency, which also points to the linkage between the *P*_e_ and error awareness. That the *N*_e_ and *P*_e_ reflect functionally dissociable error monitoring systems is further substantiated by studies demonstrating a varying dependence on dopaminergic neurotransmission (Overbeek et al., 2005): Only the *N*_e_ varies with moderate doses of alcohol (Ridderinkhof et al., 2002), benzodiazepines, and amphetamines (de Bruijn, Hulstijn, Verkes, Ruigt, & Sabbe, 2004). Likewise, mental and neurological disorders and individual differences associated with dopaminergic dysregulation affect the *N*_e_ but not – or only to a small extent – the *P*_e_ (for review, see Overbeek et al., 2005). Because of their specific functional significance, both components appear to be ideal for investigating the question if highly narcissistic individuals are vigilant towards their self-caused errors and activate a variety of defensive responses (possibly reflected in higher *N*_e_ amplitudes) or if they cognitively avoid their errors to safeguard their mentally represented grandiosity (possibly reflected in lower *P*_e_ amplitudes).

### 1.2 Objectives and hypotheses

So far, narcissism-related variations in the *N*_e_ and *P*_e_ have not been studied; however, the review of the available literature allowed us to derive distinct hypotheses. First, we expected that Rivalry is negatively related to *N*_e_ amplitudes (higher Rivalry, more negative *N*_e_) as the defensive nature of this narcissism facet can easily be related to the concept of trait defensive reactivity (Weinberg et al., 2012). Second, we hypothesised that higher admiration was linked to lower *P*_e_ amplitudes, possibly resulting from decreased error awareness (Nieuwenhuis et al., 2001). The mentally represented grandiosity of individuals with high admiration (Back et al., 2013) should be inconsistent with conscious error recognition. Such inconsistency would impair mental functioning (Grawe, 2004), wherefore individuals with a strong mental representation of their grandiosity should inhibit conscious error recognition. This hypothesis parallels the finding that individuals with high admiration inhibited processing of their own face presumably to prevent evidence from reaching consciousness that potentially contradicts one’s conviction of looking highly attractive (Mück et al., 2020). The assumption of a reduced conscious error recognition is in line with the postulation by Horvath and Morf (2009) that repression – i.e. the automatic, unconscious defence that prevents (ego-)threats from reaching consciousness (Erdelyi, 2006; Wegner & Zanakos, 1994) – constitutes the central self-defensive strategy in the repertoire of highly narcissistic individuals.

To test these hypotheses, participants filled in the Narcissistic Admiration and Rivalry Questionnaire (NARQ; Back et al., 2013) and performed a speeded go/no-go task that involved ego-threatening feedback. The feedback was thought to enhance the *N*_e_ for high Rivalry and reduce the *P*_e_ for high Admiration: When errors pose an alarming ego threat, amplified by ego-threatening feedback, the hypothesised neural responses should be even more pronounced.

## 2. Methods

### 2.1 Participants

A total of 89 participants (64 females, 25 males, no one identified as diverse; mean age = 24.27 years, *SD* = 6.00) right-handed students from the University of Cologne participated and received course credit for participation. None of the participants reported to have suffered from a neurological illness, and every participant had either normal or corrected-to-normal vision. The ethics committee of the German Psychological Association approved the study and participants gave written consent.

### 2.2 Psychometric assessment and cover story

Internal consistency for the Admiration scale reached a Cronbach’s α of 0.85 and for the Rivalry scale a Cronbach’s α of 0.83. Mean and standard deviation for the Admiration scale were 3.56 ± 0.82 (range: 1.55 to 5.44, centred range: −1.95 to 1.94) and for the Rivalry scale 2.28 ± 0.81 (range: 1.00 to 5.89, centred range: −1.28 to 3.61).

The participants completed the NARQ before the experimental task. This way, the experimental manipulations could not affect the psychometric data. However, the NARQ could affect the experimental data by suggesting that the study was about narcissism. To prevent the participants from guessing the study’s actual purpose and therefore disbelieving the faked (ego-threatening) performance feedback, they were told a cover story: After participants arrived at the laboratory, the experimenter asked them if they could participate in another study before the actual experiment, which a colleague would supervise. This study would contain a few questions and only last a few minutes. Participants were told that they would be compensated with the respective course credit. All of the 89 participants agreed to participate and completed the NARQ. At the end of the experiment, participants were debriefed verbally and were given a written document explaining the study’s actual background.

### 2.3 Experimental task

After completing the NARQ, participants performed a speeded go/no-go task, which highly resembled the task designed by Vocat et al. (2008), who also examined the *N*_e_ and the *P*_e_. They demonstrated that it provoked many errors, which were necessary for the statistical analyses of both components. The task in the current study was programmed with Uvariotest (Gerhard Mutz). During the task, participants sat in front of a computer screen. A chin rest was used (at a 60 cm distance to the screen) to reduce unwanted movements. The experiment was divided into two sessions. Each session comprised three blocks, separated by a short break, which lasted at least one minute and could be prolonged at will by the participant. Each block contained 96 trials – adding up to a total of 576 experimental trials. Note that the trial number was raised from 84 trials per block for the first nine participants to 96 for the following participants to increase the error frequency.

#### 2.3.1 Trial course

**Figure 1.**
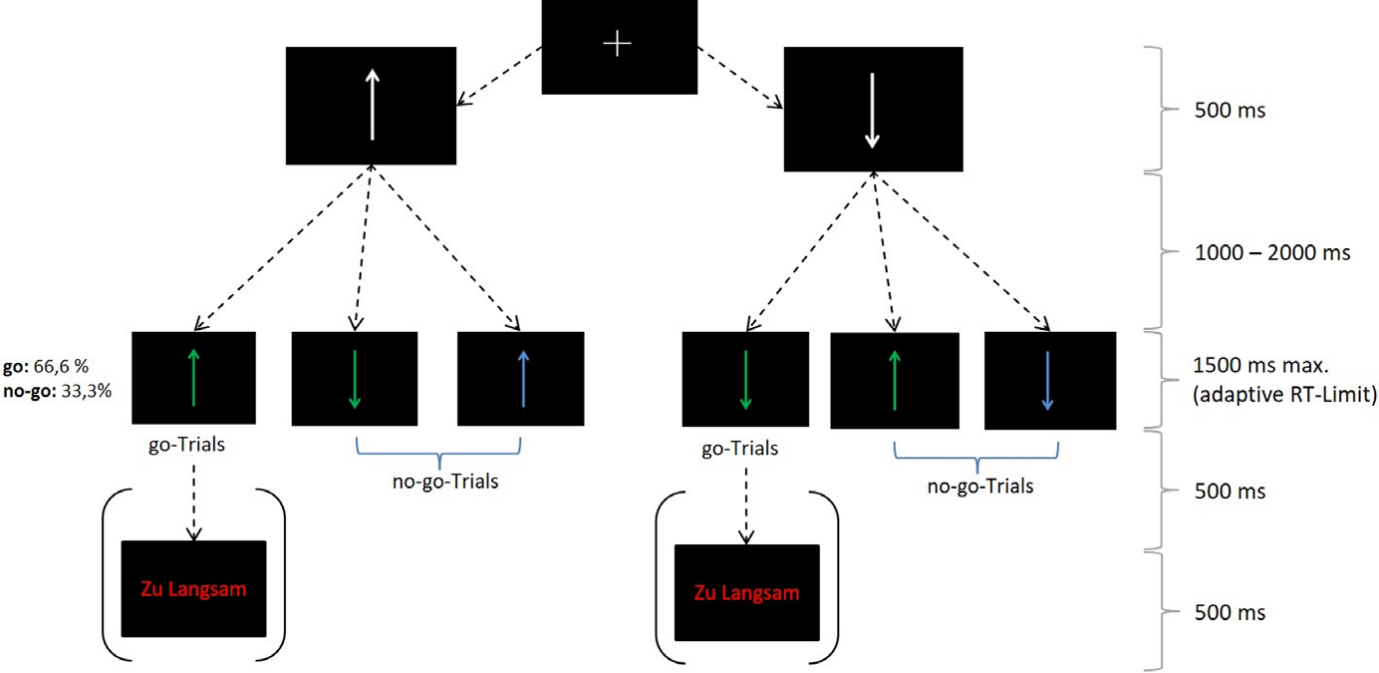
Trial design. *Note*. This figure resembles the task illustration presented by Vocat et al. (2008). It shows all possible go and no-go trials.

Each trial started with the appearance of a white fixation cross on a black screen (Figure 1). After 500 ms, a white arrow replaced the fixation cross, pointing either up- or downwards, which remained on the screen for a variable duration (1000–2000 ms). Then, the target arrow replaced this white arrow. Participants had to respond to the target arrow when it appeared in green colour *and* pointed in the same direction as the initial white arrow by pressing a key (go trials). In all other cases (when the target arrow pointed in the opposite direction of the initial white arrow and was green *or* when the arrow pointed in the same direction but was blue), the participants should withhold their response (no-go trials). When, in go trials, participants failed to respond within the RT limit, the words “Zu Langsam” (German for “Too Slow”) appeared on the screen and signalled that they had to respond faster in the subsequent go trial.

#### 2.3.2 Adaptive response time limit

Before the task, participants were given verbal and written task instructions to respond as quickly and accurately as possible. The RT limit was adjusted individually for every participant (see Vocat et al., 2008) to provoke a large number of errors and keep the task challenging despite learning effects. For Session 1, the RT limit was set to the mean RT of a calibration block (24 trials) preceding the actual experiment. Note that, before this calibration block, the participants performed another 12 practice trials to get to know the task and the apparatus. Similar to Vocat et al. (2008), the RT limit was adjusted in Session 2 to counteract possible learning effects (i.e. 95% of the mean RT in Session 1).

#### 2.3.3 Performance feedback

**Figure 2.**
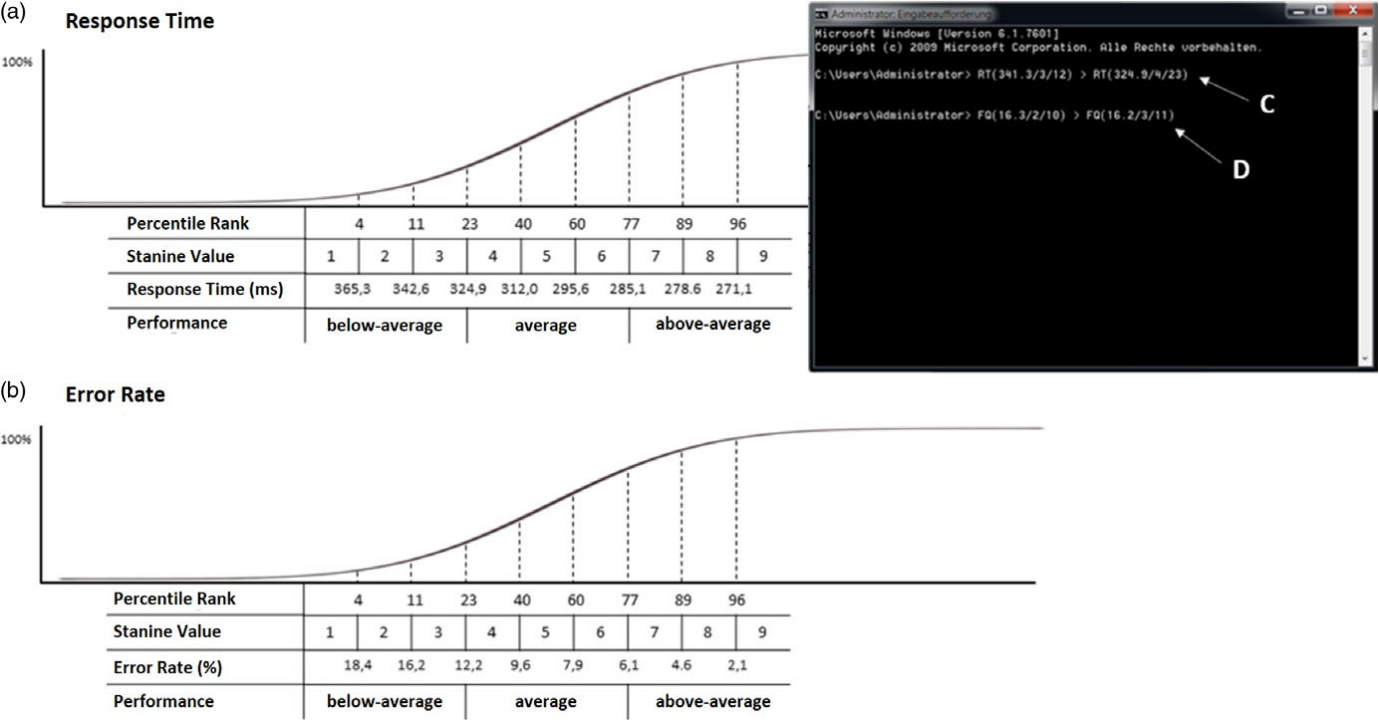
Performance feedback. *Note*. All participants were shown (false) norm values of the task for (A) RT and (B) error rate – incorporated into fictional cumulative distributions of both parameters. After explaining the rationale of stanine values, a window popped up on the screen indicating the participants’ (faked) performance data. Total values, stanine values, and percentile ranks of the RT (C) and the error rate (D) were presented, and participants were instructed to improve performance by one stanine value in both parameters. Originally, the figure was presented in German.

Following Session 1, an ego-threatening situation was created by presenting participants an unexpected (faked) feedback about their performance, creating a situation of relevance for highly narcissistic individuals (Hardaker et al., 2019). Participants were told that the task is usually used to assess concentration. However, in the current study, this task would be used to measure the influence of motivation on action monitoring ERP components. To measure the impact of motivation on the ERP components, they should improve their performance regarding their RT and error rate in the second session. To this end, the experimenter showed participants (fictional) norm values for the task – comprising (faked) total values, stanine values, and percentile ranks for RT and error rate data. These norm values were incorporated into a figure that indicated (fictional) cumulative distributions (Figure 2A and 2B). After instructing participants that they should improve their performance by one stanine value (to ensure that participants understood the rationale of stanine values, these were explained by referring to Figure 2), a window appeared on the screen displaying their (faked) relatively poor performance. The appearing data indicated that participants’ RT data corresponded to a stanine value of 3 (Figure 2C), and their error rate reflected a stanine value of 2 (Figure 2D). Participants were instructed to improve their performance regarding both parameters by one stanine value. Thus, they were told that they performed poorly and were instructed to respond faster *and* more accurately. Of course, the debriefing contained information on this faked feedback.

#### 2.3.4 Apparatus

To record behavioural data, we used a set of eight custom-made force-sensitive keys (see also Stahl et al., 2020). In the current study, we only used one of the eight response keys, namely the key on which the right index finger rested. A force sensor embedded in this key (FCC221-0010-L, DigiKey MSP6948-ND) continuously registered the force applied by the index finger. The key was calibrated prior to the experiment so that the weight of the participant’s finger functioned as the baseline for force registration. The analogous response signal was digitised by a VarioLab Ad converter (Becker-Meditec) at a sampling rate of 1024 Hz with a resolution of 16 bits. A brightness-sensitive photo sensor attached to the screen captured the near real-time stimulus onset.

### 2.4 Data acquisition

#### 2.4.1 Response Time Data

RT was calculated as the time span between stimulus onset and the point in time at which response force exceeded 50 cN (Drizinsky et al., 2016; Stahl et al., 2015).

#### 2.4.2 Electrophysiological recording, pre-processing, and data analysis

The active Ag/AgCl electrodes (Brain Products, Germany) were set up according to the international 10–20 system (FP1, FP2, F7, F3, Fz, F4, F8, FC5, FC1, FC2, FC6, T7, C3, Cz, C4, T8, CP5, CP1, CP2, CP6, P7, P3, Pz, P4, P8, FCz, O1, Oz, O2, AF7, AF3, AF4, AF8, F5, F1, F2, F6, C3’, FT7, FC3, FC4, FT8, C4’, C5, C1, C2, C6, TP7, CP3, CPz, CP4, TP8, P5, P1, P2, P6, PO7, PO3, POz, PO4, PO8; Jasper, 1958) and online referenced to the left mastoid; the right mastoid served as a passive reference. Vertical and horizontal electrooculograms (EOG) were derived from two electrodes above and below the left eye and two electrodes located beside the outer left and right canthi. The electrophysiological data were recorded with the *BrainAmp Vision Recorder* (Brain Products), while electrode impedances were kept below 10 kΩ. The EEG signal was digitised at a sampling rate of 500 Hz with a BrainAmp DC amplifier (Brain Products) and filtered online using a low-pass filter with a cut-off frequency at 70 Hz and a notch filter at line frequency (50 Hz).

Data were divided into segments ranging from 100 ms before, to 600 ms after response onset. A first artefact rejection was applied with a criterion of ±900 μV to eliminate bad epochs, and an occular correction computationally removed blinks (Gratton et al., 1983). A baseline correction, which started 100 ms before response onset, was followed by a second artefact rejection with a criterion of ±100 μV. The EEG data were averaged separately for errors and correct responses (Response Type), and Session 1 and Session 2 (Session Type). We transformed the EEG signals with a current source density (CSD) analysis, which counteracted overlapping, no-process related activity from adjacent electrode sites and made the EEG signals independent from the references (Perrin, Pernier, Bertrand, & Echalli, 1989).

*N*_e_ peak amplitudes were measured at electrode site FCz from 0 to 150 ms after response onset and *P*_e_ peak amplitudes were assessed at electrode site Cz from 150 to 300 ms after response onset (analogously to Stahl et al., 2015). Two participants committed less than six errors in one session. As the *N*_e_ and the *P*_e_ can only be accurately quantified with at least six error trials (Pontifex et al., 2010), these participants had to be excluded from the ERP analyses.

### 2.5 Statistical analyses

Multilevel models (Baayen et al., 2008) were calculated to assess the effects of the within-subject factors (Response Type and Session Type) and the NARQ scales (Admiration and Rivalry) on the dependent variables of interest (*N*_e_ and *P*_e_). Maximum likelihood estimation determined the parameters of the calculated multilevel models (Twisk, 2006). Participants were included as a random-effects variable, allowing intercepts in the dependent variables to vary between participants. This improved model fit of the multilevel models analysing effects on RT (*SD* = 21.06 ms [95% CI: 14.89 ms, 29.79 ms], χ^2^ (1) = 10.30, *p* = .001), *N*_e_ (*SD* = 0.018 µV/cm^2^ [95% CI: 0.015 µV/cm², 0.020 µV/cm²], χ^2^ (1) = 245.99, *p* < .0001), and *P*_e_ (*SD* = 0.020 µV/cm² [95% CI: 0.017 µV/cm², 0.024 µV/cm²], χ^2^ (1) = 60.94, *p* < .0001). Crucially, the multilevel models respected the nested structure of the data: Two within-subject factors (Response Type and Session Type) were investigated within each participant.

In a first step, the within-subject factors Response Type (dummy-coded: hits = 0, errors = 1) and Session Type (Session 1 = 0, Session 2 = 1), as well as their interaction were entered into the multilevel models to test general effects on the dependent variables – apart from the effects of both NARQ scales. The factor Response Type enabled the comparison between erroneous (Errors and Too-Slow Errors) and correct responses (Hits and Too-Slow Hits). We did not differentiate between Colour and Orientation Errors because many participants did not commit enough Colour Errors for such a comparison (less than six, see Pontifex et al., 2010); Vocat et al. (2008) also pooled together both error types for the analyses of the *N*_e_ and the *P*_e_. The factor Session Type allowed for comparing the dependent variables between Session 1 and Session 2. Additionally, in the multilevel models for the *N*_e_ and the *P*_e_, the total number of errors (centred) was entered as a predictor into the model to test for confounding effects of this variable.

In a second step, the continuous predictors Admiration and Rivalry and all possible interaction terms were entered in the multilevel models. The NARQ subscales were centred (Aiken & West, 1991) and the analyses were run with the *R*-package *nlme* (Pinheiro et al., 2010). To disentangle interaction effects that involved any of the narcissism scales, we applied the Johnson-Neyman technique (Johnson & Fay, 1950; Johnson & Neyman, 1936) using the *R*-package *interactions* (Long, 2021). This technique allows to identify values of a continuous moderator for which a continuous or categorical predictor of interest has a significant effect on the dependent variable. The range of these values of the moderator is termed the Johnson-Neyman interval.

## 3. Results

### 3.1 Response frequencies

In go trials, 97.38 ± 0.51% hits (mean percentage ± standard error in percentage) and 2.62 ± 0.51% misses occurred across both sessions. Of these hits, 54.27 ± 1.63% were executed within the individual RT limit. Table 1 presents response type frequencies in go trials, separated by sessions.

**Table 1.** Response type frequencies in go trials

Response Type	Session 1			Session 2		
	***M*** **in %**	***SE*** **in %**	***n*** **(min, max)**	***M*** **in %**	***SE*** **in %**	***n*** **(min, max)**
Hits	98.82	0.43	187.34 (127, 192)	95.94	0.80	181.92 (119, 192)
Fast Hits	60.89	1.66	115.68 (48, 168)	44.88	1.81	85.38 (7, 154)
Too-Slow Hits	37.93	1.58	71.67 (20, 142)	51.07	1.85	96.54 (38, 175)
Misses	1.18	0.43	2.26 (0, 65)	4.06	0.80	7.68 (0, 73)

*Note*. For the first nine participants, each session contained 36 fewer trials than for the following participants. *M* = mean, *SE* = standard error, *n* = total number of Response Type.

Error commission rate in no-go trials was 31.67 ± 1.64% across both sessions. On average, participants committed 59.72 errors in both sessions. Of these errors, 80.80 ± 1.36% were orientation errors, and the other 19.20 ± 1.36% were colour errors. In total, 77.09 ± 1.40% of errors occurred within the individual RT limit, and 22.91 ± 1.40% of errors exceeded the RT limit. Table 2 shows frequencies of the specific response types occurring in no-go trials for each session.

**Table 2.** Response Type frequencies in no-go trials

Response Type	Session 1			Session 2		
	***M*** **in %**	***SE*** **in %**	***n*** **(min, max)**	***M*** **in %**	***SE*** **in %**	***n*** **(min, max)**
Errors	27.48	1.61	25.89 (5, 79)	35.85	1.83	33.83 (4, 77)
Colour-Errors	5.54	0.88	5.13 (0, 42)	9.61	1.02	9.00 (0, 35)
Orientation Errors	21.94	0.96	20.76 (4, 41)	26.23	1.04	24.84 (0, 44)
Fast Errors	21.27	1.18	20.10 (3, 52)	27.08	1.47	25.64 (1, 65)
Slow Errors	6.21	0.73	5.79 (0, 39)	8.77	0.76	8.19 (0, 35)
Correct Rejections	72.52	1.61	68.91 (5, 91)	64.15	1.83	60.97 (17, 91)

*Note*. For the first nine participants, each session contained 36 fewer trials than for the following participants. *M* = mean, *SE* = standard error, *n* = total number of Response Type, Fast Errors = errors within the RT limit, Slow Errors = errors exceeding the RT limit.

### 3.2 Response times

RTs for the different response types and sessions are presented in Table 3. Note that this table depicts RTs for fast responses (within the RT limit) and “Too-Slow” responses separately.

**Table 3.** Response times

Response Type	Session 1			Session 2		
	*M* (ms)	***SE*** **(ms)**	***SD*** **(ms)**	***M*** **(ms)**	***SE*** **(ms)**	***SD*** **(ms)**
*Fast Responses*						
Hits	264.33	2.83	26.67	222.89	4.18	39.47
Colour Errors	225.86	4.54	37.72	188.83	4.40	38.13
Orientation Errors	242.97	2.40	22.66	209.84	3.58	33.81
*Slow Responses*						
Hits	365.83	4.87	45.97	312.34	3.63	34.20
Colour Errors	371.63	17.52	110.81	287.08	4.40	32.03
Orientation Errors	386.54	8.76	76.83	317.50	6.33	58.33

*Note*. *M* = mean, *SE* = standard error, *SD* = standard deviation, *Fast Responses* = responses within the RT limit, *Slow Responses* = responses exceeding the RT time limit.

The multilevel model for RT showed that participants responded significantly faster in Session 2 (mean ± standard error: 266.01 ± 3.40 ms) than in Session 1 (312.14 ± 4.22 ms), *b* = −46.50, *t*(599) = −6.22, *p* < .001. The difference in RTs between hits (291.35 ± 3.45 ms) and all error types (283.68 ± 4.57 ms), *b* = −8.05, *t*(599) = −1.06, *p* = .292, as well as the interaction of session type and response type was not significant, *b* = 1.05, *t*(599) = 0.10, *p* = .922. In the next step, Admiration, Rivalry, and every possible interaction term were entered into the multilevel model. Besides the effect of session type, the model did not reveal any other significant effect.

### 3.3 Event-related potentials

**Figure 3.**
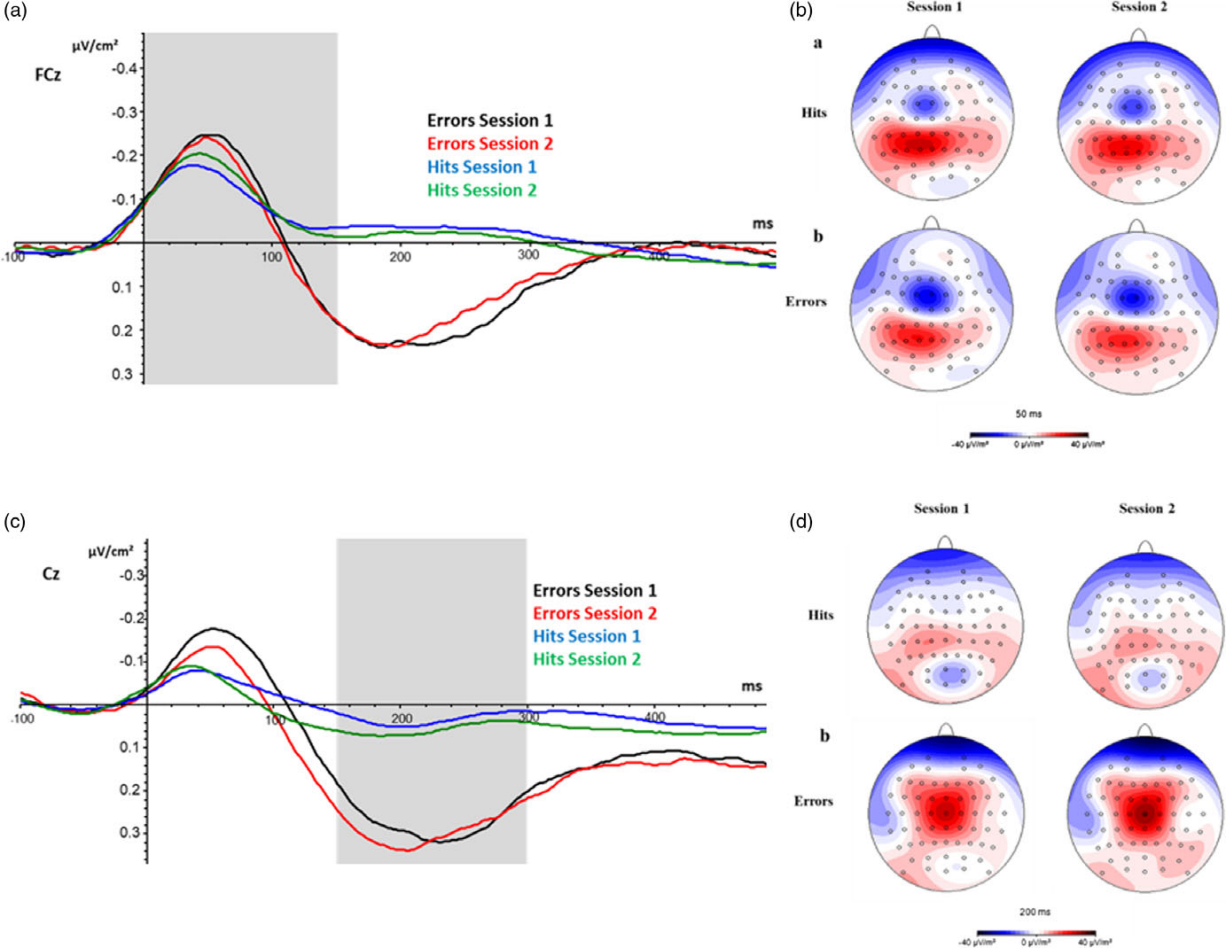
Waveforms and topographic maps for the *N*_e_ and *P*_e_ components and topographic maps of mean CSD-transformed ERPs, 50 ms and 200 ms after response onset. *Note*. (A) Response locked CSD-ERP waveforms at electrode position FCz (the grey area indicates the time window in which the *N*_e_ was inspected). (B) Topographic maps of mean CSD-transformed ERPs, 50 ms after response onset, show the *N*_e_’s negative deflection manifesting in blue colour at electrode site FCz. (C) CSD-ERP waveforms at electrode position Cz (the grey area indicates the time window in which the *P*_e_ was inspected). (D) Topographic maps of mean CSD-transformed ERPs, 200 ms after response onset, indicate (for error trials) the *P*_e_’s positive deflection manifesting in red colour at electrode site Cz.

Grand average CSD-transformed ERP waveforms showed the occurrence of a distinct *N*_e_ at electrode site FCz, 0 to 150 ms after response onset (Figure 3A). Topographic maps of mean CSD-transformed ERPs for errors and hits, in Session 1 and Session 2, highlight the characteristic location of the *N*_e_ and show a higher negative deflection for Errors than for Hits at electrode site FCz (Figure 3B). Grand average CSD-transformed ERP waveforms also showed the occurrence of a clear *P*_e_ at electrode site Cz, between 150 and 300 ms after Errors but not after Hits (Figure 3C). Topographic maps, highlight the characteristic, more diffuse location of the *P*_e_ at electrode site Cz for Errors (Figure 3D).

#### 3.3.1 The *N*_e_ and narcissism

The multilevel model analysing general effects of Response Type and Session Type indicated a significant main effect of Response Type on the *N*_e/c_ amplitude, *b* = 0.093, *t*(258) = −6.23, *p* < .001. The *N*_e_ was larger (−0.299 ± 0.017 µV/cm²) than the *N*_c_ (−0.225 ± 0.014 µV/cm²). Entering the NARQ scales and all possible interaction terms into the model resulted in the data presented in Table 4.

**Table 4.** Multilevel model assessing the predictive value of Admiration and Rivalry on the *N*_e_

	*b*	*SE b*	95% CI	***P***
Intercept	−0.210	0.023	−0.254, −0.167	<0.001***
Number of Errors	0.001	0.001	−0.003, 0.003	0.134
Session Type	−0.025	0.015	−0.054, 0.004	0.103
Response Type	−0.097	0.015	−0.126, −0.673	<0.001***
Admiration	0.044	0.029	−0.012, 0.101	0.133
Rivalry	0.006	0.032	−0.057, 0.068	0.858
Admiration × Rivalry	−0.027	0.026	−0.078, 0.025	0.315
Sessions Type × Response Type	0.038	0.022	−0.003, 0.080	0.076
Session Type × Admiration	−0.022	0.019	−0.059, 0.015	0.250
Session Type × Rivalry	−0.016	0.022	−0.058, 0.025	0.451
Response Type × Admiration	−0.003	0.019	−0.040, 0.034	0.878
Response Type × Rivalry	−0.047	0.022	−0.088, −0.005	0.031*
Response Type × Admiration × Rivalry	0.018	0.018	−0.016, 0.052	0.312
Session Type × Admiration × Rivalry	0.026	0.018	−0.008, 0.060	0.149
Session Type × Response Type × Admiration	0.001	0.027	−0.051, 0.053	0.969
Session Type × Response Type × Rivalry	0.015	0.031	−0.044, 0.074	0.629
Session Type × Response Type × Admiration × Rivalry	−0.007	0.025	−0.055, 0.041	0.772

* *P* < .05,

*** *P* < .001.

**Figure 4.**
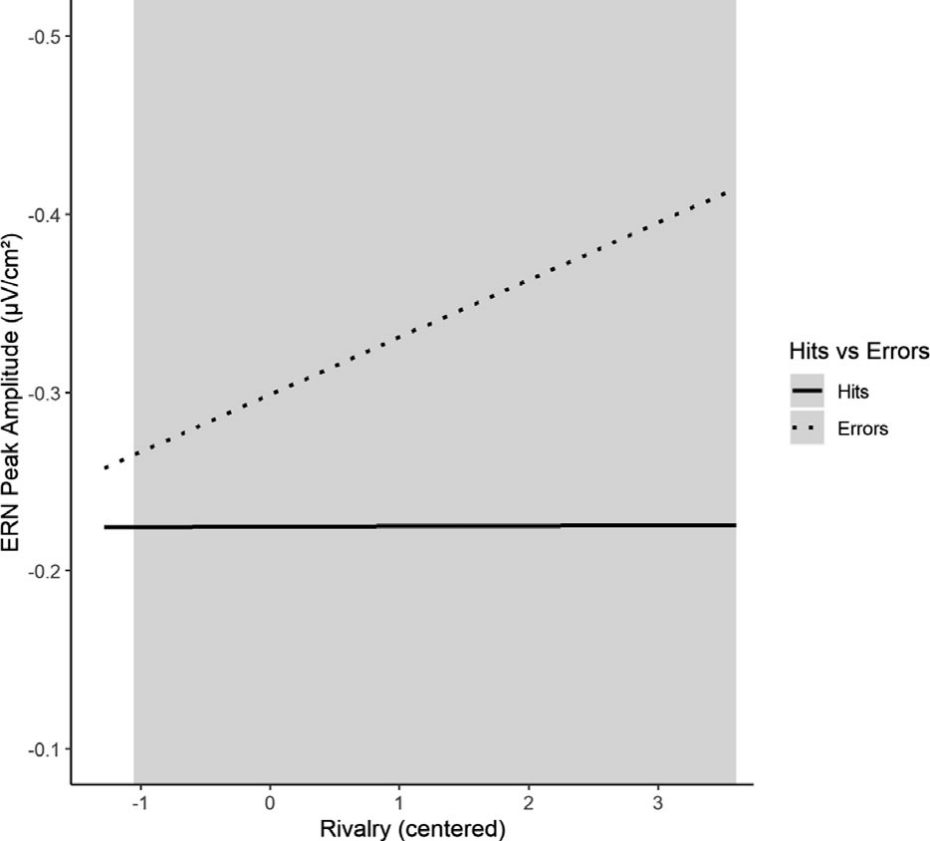
Interaction effect of Rivalry with Response Type on the *N*_e_ amplitude. *Note*. The grey area indicates the regions of significance for this interaction effect. The interaction effect is illustrated (only) for the range of the observed centred Rivalry scores (min = −1.28, max = 3.61).

In addition to the main effect of Response Type, the model showed a significant interaction effect of Rivalry and Response Type on the *N*_e_. The Johnson–Neyman technique indicated that *N*_e_ differences between Hits and Errors were significant for all Rivalry scores >−1.06. Increasing Rivalry scores were associated with an increasing N_e_ − *N*_c_ amplitude difference (Figure 4).

#### 3.3.2 The *P*_e_ and narcissism

The multilevel model analysing general effects on the *P*_e_ indicated a significant effect of Response Type: Hits were associated with a lower *P*_e_ (0.095 ± 0.014 µV/cm²) than Errors (0.389 ± 0.024 µV/cm²), *b* = 0.300, *t*(258) = 12.34, *p* < .001. The multilevel model, including the NARQ scales and all possible interaction terms, indicated no other significant effects on the *P*_e_. The results of this model are presented in the supplement.

## 4. Discussion

For the first time, the current study investigated variations of narcissism with error processing on a neural level. With a speeded go/no-go task, we demonstrated that participants with higher Rivalry displayed higher *N*_e_ amplitudes after errors. This finding serves as primary evidence that specific responses to failures in narcissism (usually observed at later processing stages, i.e. at the behavioural and self-report level) also occur in early neural processes involved in error processing. In contrast to our predictions, Admiration did not vary with the *P*_e,_ and the performance feedback (intended to create an even more ego-threatening situation) did not moderate any effects on the *N*_e_ or *P*_e_.

### 4.1 Rivalry and vigilance towards errors

Our data indicated that participants with high Rivalry showed enhanced processing of performance errors at an early processing stage: the higher Rivalry, the higher the difference between the *N*_e_ (negativity in error trials) and the *N*_c_ (negativity in correct trials). For participants with very low Rivalry, we could not observe any difference between the *N*_e_ and the *N*_c_. We assumed that the *N*_e_ indicates vigilance to failure in narcissism. Accordingly, the data point to higher vigilance to failures with higher Rivalry. This interpretation is in line with a systematic review of 34 neuroscience studies highlighting that, in response to self-relevant stimuli such as stimuli indicating social exclusion (Cascio et al., 2015), narcissism is linked to increased autonomic, neuroendocrine, and neurophysiological stress reactions (Jauk & Kanske, 2021). These stress reactions manifest, for example, in higher systolic blood pressure (Sommer et al., 2009), cortisol levels (Edelstein et al., 2010), and salience network activation (Cascio et al., 2015; Jauk et al., 2017). However, such stress reactions in narcissism have only been observed for self-relevant stimuli (Jauk & Kanske, 2021). Stimuli that are stressful and self-related but not self-relevant, like loud noises (Kelsey et al., 2001), or other-related stimuli (Fan et al., 2011; Scalabrini et al., 2017) do not elicit such stress reactions or even lead to down-regulation in the corresponding systems (Jauk & Kanske, 2021). Jauk and Kanske (2021) concluded, in line with our assumption, that grandiose narcissism is linked to a heightened vigilance but only in the context of self-relevant and therefore potentially ego-threatening stimuli. The current ERP data, which are time-locked to (self-relevant) errors, are thus in line with several neuroscientific studies already conducted in this line of research.

Beyond interpreting the error-specific activity reflected in the *N*_e_ as an indicator of vigilance, Weinberg et al. (2012) considered the *N*_e_ as a reflection of one’s *trait defensive reactivity*. Accordingly, high Rivalry seems to be associated with this heightened dispositional tendency to immediately initiate several defensive responses after endogenous threats, e.g. after errors. With this higher early error monitoring activity, high Rivalry participants might enhance cognitive control to improve their performance (Holroyd & Yeung, 2012; Mattes et al., 2022b; Ridderinkhof et al., 2004) – and thereby protect their grandiosity. Also, they might recruit more affective and motivational resources after errors (e.g. Amodio et al., 2008; Pourtois et al., 2010) to energise their self-protection in this potentially ego-threatening situation. A heightened trait defensive reactivity could generally help individuals with high Rivalry to quickly adjust their experience and behaviour to ego threats in a way that protects their grandiosity. Exactly this preoccupation with protecting oneself against ego threats was described for the Rivalry pathway (Back et al., 2013). Yet, it is noteworthy that this self-protection is not only described on a conceptual level but seems to occur at very early information processing stages, within 150 ms after error commission, and can be measured on a neural level.

Interestingly, only for the lowest Rivalry scores (centred scores ≤ −1.06), the *N*_e_ was *not* significantly higher for errors than the *N*_c_ for correct responses. One could conclude that participants with very low Rivalry scores do not process errors at this early perceptual stage more intensely than correct responses. Possibly, these participants did not activate defensive resources to counteract an error (by recruiting additional cognitive, affective, and motivational resources) because they did not perceive an error as (ego-)threatening. One can speculate that individuals with low Rivalry are not afraid of experiencing vulnerability and imperfection and, thus, do not boost their error processing reflected in the *N*_e_.

Rivalry emerged in the current study as another trait variable that varies with the *N*_e_. which fits easily together with findings on variations between the *N*_e_ and other variables related to Rivalry. For example, a pronounced Behavioural Inhibition System (Amodio et al., 2008) and a competitive context (García Alanis et al., 2019) were also demonstrated to be linked to higher N_e_ amplitudes. The Behavioural Inhibition System was shown to positively correlate with Rivalry, and competing with others seems to be a key aspect of Rivalry (Back et al., 2013).

### 4.2 Weaker conscious awareness of self-caused errors in narcissism?

The second hypothesis that Admiration is linked to a lower P_e_, an ERP component indicating error awareness (Overbeek et al., 2005), could not be confirmed. The literature suggested that highly narcissistic individuals are less aware of their failures and imperfection in everyday life situations (Campbell et al., 2004; Hardaker et al., 2019; Horvath & Morf, 2009; Kernis & Sun, 1994; Liu et al., 2019). We assumed that their reduced awareness of imperfection, indicating repression (Erdelyi, 2006; Horvath & Morf, 2009), might be accompanied by reduced error awareness reflected in smaller *P*_e_ amplitudes. Especially, individuals with high Admiration should show smaller *P*_e_ amplitudes, as errors are inconsistent with their consciously represented grandiosity and would impair consistent mental functioning (Grawe, 2004). However, our results did not show a lower *P*_e_ for higher Admiration. One could conclude that participants with high Admiration are as aware of their self-caused errors as others (Overbeek et al., 2005) but this conclusion appears premature when considering the error evidence accumulation account, which suggests that the *P*_e_ merely reflects the amount of accumulated error evidence in a decision process that can *potentially* lead to error awareness (Steinhauser & Yeung, 2010, 2012). When error evidence accumulation reaches a certain threshold, the participant becomes aware of the error and is able to signal error commission (Steinhauser & Yeung, 2010, 2012). In line with this, Boldt and Yeung (2015) reported that the *P*_e_ varies with gradual changes in decision confidence (expressed on a 6-point scale ranging from “certainly wrong” to “certainly correct”) being highest for “certainly wrong” and reducing gradually for the other subjective ratings (Boldt & Yeung, 2015). According to this literature, the *P*_e_ reflects error evidence accumulation and not error awareness itself (Steinhauser & Yeung, 2010, 2012). In light of this literature, our data indicate that individuals with high Admiration accumulate as much error evidence as individuals with low Admiration (reflected in similar *P*_e_ amplitudes). Yet, they might have a higher internal decision criterion at which an error is consciously detected. Casually worded, individuals with high Admiration scores possibly need to be confronted with more and clearer error evidence until they admit to themselves and others that they have committed an error. However, our data cannot substantiate this assumption, and – based on our findings – more specific studies can be designed.

### 4.3 Future studies and limitations

To examine if the decision criterion at which an error can be consciously reported (Steinhauser and Yeung, 2010, 2012) varies with Admiration, future studies could use an error signalling paradigm in which participants index their response confidence (Rabbitt, 1968, 2002; for ERPs, see Boldt & Yeung, 2015). One can hypothesise that Admiration moderates the effect of error signalling behaviour on the *P*_e_. That is, individuals with high Admiration might show higher *P*_e_ amplitudes for signalled errors.

Moreover, it would be interesting to link error detection itself to incentives – not the performance in a primary task. A paradigm in which participants would be rewarded for a high error detection accuracy possibly circumvents the self-enhancing bias in narcissism (Raskin et al., 1991). In such a task, errors would still be ego-threatening, but highly narcissistic individuals would nevertheless be eager to accurately detect their errors when this would be framed as a sign of their grandiosity. Thereby, one might better understand the variations of narcissism, error signalling, and the *P*_e_ with less confounding effects by the self-enhancing bias in narcissism (Raskin et al., 1991).

Also, it would be interesting to study variations of narcissism with another error processing ERP component: the feedback-related negativity (FRN; Hauser et al., 2014; Miltner et al., 1997; Nieuwenhuis et al., 2004). Miltner et al. (1997) demonstrated that not only an incorrect response elicits a negative deflection in form of the *N*_e_ but also trial-by-trial *feedback* indicating a false response. This FRN peaks within 200 to 300 ms after stimulus onset at mid-central electrode sites and is computed as the wave difference between feedback indicating a false and correct response (Hauser et al., 2014; Nieuwenhuis et al., 2004). Neural responses to feedback could vary with narcissism, not least because narcissism-specific responses to feedback have already been demonstrated on an explicit level: Kernis and Sun (1994) reported that highly narcissistic individuals attributed more (less) competence to the diagnostician and a higher (lower) diagnosticity to the evaluation technique when receiving positive (negative) feedback on a given speech compared to individuals with low narcissism. Such varying explicit responses to feedback could also manifest on a neural level, in FRN variations. It has to be noted that variations of narcissism with the FRN have already been examined in a monetary gambling task with low- and high-risk decisions (Yang et al., 2018a) and in an ultimatum game in which participants were given fair and unfair offers (Yang et al., 2018b). Neither of these studies demonstrated variations between narcissism and the FRN but neither investigated the FRN in response to self-caused action errors.

We were interested in the question of whether Admiration and Rivalry, two central strategies to maintain narcissistic grandiosity, are linked to error-specific brain activity: We hypothesised that Admiration is related to cognitive avoidance and Rivalry to hypervigilance to self-caused failures, which should affect error processing ERP components. To clarify, Admiration and Rivalry only capture aspects of narcissism related to grandiosity and self-entitlement; the NARC does not aim to assess vulnerable aspects of narcissism (Krizan & Herlache, 2018). Thus, future studies could also investigate variations of error processing ERP components with vulnerable narcissism. One can also assume higher *N*_e_ amplitudes for vulnerability given that higher *N*_e_ amplitudes were found for related constructs like higher self-reported negative affect (Hajcak et al., 2004; Luu et al., 2000), higher worries, and higher general anxiety (Hajcak et al., 2003).

The paradigm was constructed to establish ego-threatening conditions. For this reason, an ego-threatening feedback was implemented – after the first half of the experiment – to show participants that they had performed poorly in Session 1 and to urge them to perform better in Session 2. The results showed that this (faked) ego-threatening feedback neither affected the *N*_e_ nor the *P*_e_ and neither covaried with Admiration nor with Rivalry. This lack of effect could be explained by the potentially high stress level that was associated with the speeded go/no-go task itself (Vocat et al., 2008). The time pressure and the high error rate in the task could have created considerable ego-threatening conditions already in Session 1 – resulting in only a minor, statistically insignificant incremental ego-threatening effect of the faked feedback. It was difficult to verify whether participants believed in the feedback. Directly asking a question about the validity of the presented feedback would have led to answers certainly confounded by the participants’ narcissism scores as highly narcissistic individuals attribute bad performances more strongly to external causes (Kernis & Sun, 1994). Hence, participants were only indirectly asked about their experiences with the experimental task settings and none of them questioned the validity of the feedback on one’s own accord.

## 5. Conclusion

At the beginning of the current study, we outlined that the literature on narcissism has suggested two contradictory ways how highly narcissistic individuals deal with their failures: either by consciously avoiding them or by vigilantly turning towards them. We suggested that this contradiction might be solved by respecting different narcissism dimensions, i.e. Admiration and Rivalry, and by taking the temporal dynamics of perceptual processing into account. The current results only supported the vigilance hypothesis: The results showed that Rivalry was linked to an intense early error processing (reflected in higher *N*_e_ amplitudes), which we interpreted as hypervigilance to self-caused failures.

## Supplementary material

For supplementary material accompanying this paper visit https://doi.org/10.1017/pen.2022.7. click here to view supplementary material

## References

[ref1] Aiken, L. S. , & West, S. G. (1991). Multiple regression: Testing and interpreting interactions. Newbury Park, London: SAGE.

[ref2] Akhtar, S. , & Thomson, J. A. Jr. (1982). Overview: Narcissistic personality disorder. The American Journal of Psychiatry, 139, 12–20. 10.1176/ajp.139.1.12 7034551

[ref3] Amodio, D. M. , Bartholow, B. D. , & Ito, T. A. (2014). Tracking the dynamics of the social brain: ERP approaches for social cognitive and affective neuroscience. Social Cognitive and Affective Neuroscience, 9, 385–393. 10.1093/scan/nst177 24319116 PMC3980796

[ref4] Amodio, D. M. , Master, S. L. , Yee, C. M. , & Taylor, S. E. (2008). Neurocognitive components of the behavioral inhibition and activation systems: Implications for theories of self-regulation. Psychophysiology, 45, 11–19. 10.1111/j.1469-8986.2007.00609.x 17910730

[ref5] Baayen, R. H. , Davidson, D. J. , & Bates, D. M. (2008). Mixed-effects modeling with crossed random effects for subjects and items. Journal of Memory and Language, 59, 390–412. 10.1016/j.jml.2007.12.005

[ref6] Back, M. D. , Küfner, A. C. P. , Dufner, M. , Gerlach, T. M. , Rauthmann, J. F. & Denissen, J. J. A. (2013). Narcissistic admiration and rivalry: Disentangling the bright and dark sides of narcissism. Journal of Personality and Social Psychology, 105, 1013–1037. 10.1037/a0034431 24128186

[ref7] Boldt, A. , & Yeung, N. (2015). Shared neural markers of decision confidence and error detection. The Journal of Neuroscience, 35, 3478–3484. 10.1523/JNEUROSCI.0797-14.2015 25716847 PMC4339357

[ref8] Botvinick, M. M. , Braver, T. S. , Barch, D. M. , Carter, C. S. , & Cohen, J. D. (2001). Conflict monitoring and cognitive control. Psychological Review, 108, 624–652. 10.1037/0033-295x.108.3.624 11488380

[ref9] Campbell, W. K. , Goodie, A. S. , & Foster, J. D. (2004). Narcissism, confidence, and risk attitude. Journal of Behavioral Decision Making, 17, 297–311. 10.1002/bdm.475

[ref10] Cascio, C. N. , Konrath, S. H. , & Falk, E. B. (2015). Narcissists’ social pain seen only in the brain. Social Cognitive and Affective Neuroscience, 10, 335–341. 10.1093/scan/nsu072 24860084 PMC4350489

[ref11] de Bruijn, E. R. , Hulstijn, W. , Verkes, R. J. , Ruigt, G. S. , & Sabbe, B. G. (2004). Drug-induced stimulation and suppression of action monitoring in healthy volunteers. Psychopharmacology, 177, 151–160. 10.1007/s00213-004-1915-6 15578258

[ref12] Debener, S. , Ullsperger, M. , Siegel, M. , Fiehler, K. , von Cramon, D. Y. , & Engel, A. K. (2005). Trial-by-trial coupling of concurrent electroencephalogram and functional magnetic resonance imaging identifies the dynamics of performance monitoring. The Journal of Neuroscience, 25, 11730–11737. 10.1523/JNEUROSCI.3286-05.2005 16354931 PMC6726024

[ref13] Dehaene, S. , Posner, M. I. , & Tucker, D. M. (1994). Localization of a neural system for error detection and compensation. Psychological Science, 5, 303–305. 10.1111/j.1467-9280.1994.tb00630.x

[ref14] Di Gregorio, F. , Maier, M. E. , & Steinhauser, M. (2018). Errors can elicit an error positivity in the absence of an error negativity: Evidence for independent systems of human error monitoring. NeuroImage, 172, 427–436. 10.1016/j.neuroimage.2018.01.081 29409999

[ref15] Di Sarno, M. , Di Pierro, R. , & Madeddu, F. (2018). The relevance of neuroscience for the investigation of narcissism: A review of current studies. Clinical Neuropsychiatry: Journal of Treatment Evaluation, 15, 242–250.

[ref16] Drizinsky, J. , Zülch, J. , Gibbons, H. , & Stahl, J. (2016). How personal standards perfectionism and evaluative concerns perfectionism affect the error positivity and post-error behavior with varying stimulus visibility. Cognitive, Affective & Behavioral Neuroscience, 16, 876–887. 10.3758/s13415-016-0438-z 27250616

[ref17] Edelstein, R. S. , Yim, I. S. , & Quas, J. A. (2010). Narcissism predicts heightened cortisol reactivity to a psychosocial stressor in men. Journal of Research in Personality, 44, 565–572. 10.1016/j.jrp.2010.06.008 21076653 PMC2976540

[ref18] Endrass, T. , Riesel, A. , Kathmann, N. , & Buhlmann, U. (2014). Performance monitoring in obsessive-compulsive disorder and social anxiety disorder. Journal of Abnormal Psychology, 123, 705–714. 10.1037/abn0000012 25286372

[ref19] Endrass, T. , Schuermann, B. , Kaufmann, C. , Spielberg, R. , Kniesche, R. , & Kathmann, N. (2010). Performance monitoring and error significance in patients with obsessive-compulsive disorder. Biological Psychology, 84, 257–263. 10.1016/j.biopsycho.2010.02.002 20152879

[ref20] Erdelyi M. H. (2006). The unified theory of repression. The Behavioral and Brain Sciences, 29, 499–551. 10.1017/S0140525X06009113 17156548

[ref21] Falkenstein, M. , Hohnsbein, J. , & Hoormann, J. (1994). Event-related potential correlates of errors in reaction tasks. In G. Karmos , M. Molnar , V. Csepe , I. Czigler , & J. E. Desmedt (Eds.), Perspectives of event-related potentials research (pp. 287–296. (EEG Suppl. 441)). Amsterdam: Elsevier Science. 7649035

[ref22] Falkenstein, M. , Hohnsbein, J. , Hoormann, J. , & Blanke, L. (1991). Effects of crossmodal divided attention on late ERP components. II. Error processing in choice reaction tasks. Electroencephalography and Clinical Neurophysiology, 78, 447–455. 10.1016/0013-4694(91)90062-9 1712280

[ref23] Falkenstein, M. , Hoormann, J. , Christ, S. , & Hohnsbein, J. (2000). ERP components on reaction errors and their functional significance: A tutorial. Biological Psychology, 51, 87–107. 10.1016/s0301-0511(99)00031-9 10686361

[ref24] Fan, Y. , Wonneberger, C. , Enzi, B. , de Greck, M. , Ulrich, C. , Tempelmann, C. , Bogerts, B. , Doering, S. , & Northoff, G. (2011). The narcissistic self and its psychological and neural correlates: An exploratory fMRI study. Psychological Medicine, 41, 1641–1650. 10.1017/S003329171000228X 21144117

[ref25] García Alanis, J. C. , Baker, T. E. , Peper, M. , & Chavanon, M. L. (2019). Social context effects on error-related brain activity are dependent on interpersonal and achievement-related traits. Scientific Reports, 9, 1728. 10.1038/s41598-018-38417-2 30741987 PMC6370841

[ref26] Gehring, W. J. , Goss, B. , Coles, M. G. , Meyer, D. E. , & Donchin, E. (1993). A neural system for error detection and compensation. Psychological Science, 4, 385–390. 10.1111/j.1467-9280.1993.tb00586.x

[ref27] Gehring, W. J. , Goss, B. , Coles, M. G. , Meyer, D. E. , & Donchin, E. (2018). The error-related negativity. Perspectives on Psychological Science, 13, 200–204. 10.1177/1745691617715310 29592655

[ref28] Gehring, W. J. , Himle, J. , & Nisenson, L. G. (2000). Action-monitoring dysfunction in obsessive-compulsive disorder. Psychological Science, 11, 1–6. 10.1111/1467-9280.00206 11228836

[ref29] Geukes, K. , Nestler, S. , Hutteman, R. , Dufner, M. , Küfner, A. C. P. , Egloff, B. , Denissen, J. J. A. , & Back, M. D. (2017). Puffed-up but shaky selves: State self-esteem level and variability in narcissists. Journal of Personality and Social Psychology, 112, 769–786. 10.1037/pspp0000093 27854443

[ref30] Grapsas, S. , Brummelman, E. , Back, M. D. , & Denissen, J. (2020). The “Why” and “How” of narcissism: A process model of Narcissistic status Pursuit. Perspectives on Psychological Science, 15, 150–172. 10.1177/1745691619873350 31805811 PMC6970445

[ref31] Gratton, G. , Coles, M. G. , & Donchin, E. (1983). A new method for off-line removal of ocular artifact. Electroencephalography & Clinical Neurophysiology, 55, 468–484. 10.1016/0013-4694(83)90135-9 6187540

[ref32] Grawe, K. (2004). Psychological therapy. Seattle: Hogrefe.

[ref33] Grundler, T. O. , Cavanagh, J. F. , Figueroa, C. M. , Frank, M. J. , & Allen, J. J. (2009). Task-related dissociation in ERN amplitude as a function of obsessive-compulsive symptoms. Neuropsychologia, 47, 1978–1987. 10.1016/j.neuropsychologia.2009.03.010 19428431 PMC2680784

[ref34] Hajcak, G. , McDonald, N. , & Simons, R. F. (2003). Anxiety and error-related brain activity. Biological Psychology, 64, 77–90. 10.1016/s0301-0511(03)00103-0 14602356

[ref35] Hajcak, G. , McDonald, N. , & Simons, R. F. (2004). Error-related psychophysiology and negative affect. Brain and Cognition, 56, 189–197. 10.1016/j.bandc.2003.11.001 15518935

[ref36] Hajcak, G. , Moser, J. S. , Yeung, N. , & Simons, R. F. (2005). On the ERN and the significance of errors. Psychophysiology, 42, 151–160. 10.1111/j.1469-8986.2005.00270.x 15787852

[ref37] Hajcak, G. , & Simons, R. F. (2002). Error-related brain activity in obsessive-compulsive undergraduates. Psychiatry Research, 110, 63–72. 10.1016/s0165-1781(02)00034-3 12007594

[ref38] Hardaker, M. , Sedikides, C. , & Tsakanikos, E. (2019). Hypervigilance to self-threat: Further experimental evidence for the mask model of narcissism. Self and Identity, 20, 297–310. 10.1080/15298868.2019.1667862

[ref39] Hauser, T. U. , Iannaccone, R. , Stämpfli, P. , Drechsler, R. , Brandeis, D. , Walitza, S. , & Brem, S. (2014). The feedback-related negativity (FRN) revisited: New insights into the localization, meaning and network organization. NeuroImage, 84, 159–168. 10.1016/j.neuroimage.2013.08.028 23973408

[ref40] Hock, M. , Krohne, H. W. , & Kaiser, J. (1996). Coping dispositions and the processing of ambiguous stimuli. Journal of Personality and Social Psychology, 70, 1052–1066. 10.1037/0022-3514.70.5.1052

[ref41] Hoffmann, S. , & Falkenstein, M. (2010). Independent component analysis of erroneous and correct responses suggests online response control. Human Brain Mapping, 31, 1305–1315. 10.1002/hbm.20937 20127872 PMC6870805

[ref42] Holroyd, C. , & Coles, M. (2002). The neural basis of human error processing: Reinforcement learning, dopamine, and the error-related negativity. Psychological Review, 109, 679–709. 10.1037/0033-295X.109.4.679 12374324

[ref43] Holroyd, C. B. , Dien, J. , & Coles, M. (1998). Error-related scalp potentials elicited by hand and foot movements: Evidence for an output-independent error-processing system in humans. Neuroscience Letters, 242, 65–68. 10.1016/s0304-3940(98)00035-4 9533395

[ref44] Holroyd, C. B. , & Yeung, N. (2012). Motivation of extended behaviors by anterior cingulate cortex. Trends in Cognitive Sciences, 16, 122–128. 10.1016/j.tics.2011.12.008 22226543

[ref45] Horvath, S. & Morf, C. C. (2009). Narcissistic defensiveness: Hypervigilance and avoidance of worthlessness. Journal of Experimental Social Psychology, 45, 1252–1258. 10.1016/j.jesp.2009.07.011

[ref46] Jasper, H. H. (1958) The ten-twenty electrode system of the International Federation. Electroencephalography and Clinical Neurophysiology, 10, 371–375. 10590970

[ref47] Jauk, E. , Benedek, M. , Koschutnig, K. , Kedia, G. , & Neubauer, A. C. (2017). Self-viewing is associated with negative affect rather than reward in highly narcissistic men: An fMRI study. Scientific Reports, 7. 10.1038/s41598-017-03935-y PMC5517462 28724894

[ref48] Jauk, E. , & Kanske, P. (2021). Can neuroscience help to understand narcissism? A systematic review of an emerging field. Personality Neuroscience, 4. 10.1017/pen.2021.1 PMC8170532 34124536

[ref49] Johnson, P. O. , & Fay, L. C. (1950). The Johnson-Neyman technique, its theory and application. Psychometrika, 15, 349–367. 10.1007/bf02288864 14797902

[ref50] Johnson, P. O. , & Neyman, J. (1936). Tests of certain linear hypotheses and their application to some educational problems. Statistical Research Memoirs, 1, 57–93.

[ref51] Kaiser, J. , Barker, R. , Haenschel, C. , Baldeweg, T. , & Gruzelier, J. H. (1997). Hypnosis and event-related potential correlates of error processing in a Stroop-type paradigm: A test of the frontal hypothesis. International Journal of Psychophysiology: Official Journal of the International Organization of Psychophysiology, 27, 215–222. 10.1016/s0167-8760(97)00055-x 9451580

[ref52] Kelsey, R. M. , Ornduff, S. R. , McCann, C. M. , & Reiff, S. (2001). Psychophysiological characteristics of narcissism during active and passive coping. Psychophysiology, 38, 292–303. 10.1017/S0048577201000051 11347874

[ref53] Kernberg, O. F. (1975). Borderline conditions and pathological narcissism. New York: Aronson.

[ref54] Kernis, M. H. , & Sun, C.-R. (1994). Narcissism and reactions to interpersonal feedback. Journal of Research in Personality, 28, 4–13. 10.1006/jrpe.1994.1002

[ref55] Kohut, H. (1977). The restoration of the self. Madison: International Universities Press.

[ref110] Krizan, Z. , & Herlache, A. D. (2018). The narcissism spectrum model: A synthetic view of narcissistic personality. Personality and Social Psychology Review, 22, 3–31. 10.1177/1088868316685018 28132598

[ref56] Krusemark, E. A. , Lee, C. , & Newman, J. P. (2015). Narcissism dimensions differentially moderate selective attention to evaluative stimuli in incarcerated offenders. Personality Disorders, 6, 12–21. 10.1037/per0000087 25330183 PMC4293238

[ref57] Liu, Y. , Li, Y. , Hao, X. , Zhang, Y. (2019). Narcissism and learning from entrepreneurial failure. Journal of Business Venturing, 34, 496–512. 10.1016/j.jbusvent.2019.01.003

[ref58] Long, J. A. (2021). Interactions: Comprehensive, user-friendly toolkit for probing interactions. R package version (Version 1.1.5). Retrieved from https://CRAN.R-project.org/package=interactions.

[ref59] Luck, S. J. (2014). An introduction to the event-related potential technique. Cambridge: MIT Press.

[ref60] Luu, P. , Collins, P. , & Tucker, D. M. (2000). Mood, personality, and self-monitoring: Negative affect and emotionality in relation to frontal lobe mechanisms of error monitoring. Journal of Experimental Psychology. General, 129, 43–60. 10.1037//0096-3445.129.1.43 10756486

[ref61] Luu, P. , Tucker, D. M. , & Makeig, S. (2004). Frontal midline theta and the error-related negativity: Neurophysiological mechanisms of action regulation. Clinical Neurophysiology, 115, 1821–1835. 10.1016/j.clinph.2004.03.031 15261861

[ref62] Marchlewska, M. , & Cichocka, A. (2017). An autobiographical gateway: Narcissists avoid first-person visual perspective while retrieving self-threatening memories. Journal of Experimental Social Psychology, 68, 157–161. 10.1016/j.jesp.2016.06.003

[ref63] Mattes, A. , Mück, M. , Stahl, J. (2022a). Perfectionism-related variations in error processing in a complex decision task. Personality Neuroscience, 5, e12. 10.1017/pen.2022.3 PMC9880962 36721395

[ref64] Mattes, A. , Porth, E. , & Stahl, J. (2022b). Linking neurophysiological processes of action monitoring to post-response speed-accuracy adjustments in a neuro-cognitive diffusion model. NeuroImage, 247, Article 118798. 10.1016/j.neuroimage.2021.118798 34896290

[ref66] Miller, J. D. , Lynam, D. R. , Hyatt, C. S. , & Campbell, W. K. (2017). Controversies in narcissism. Annual Review of Clinical Psychology, 13, 291–315. 10.1146/annurev-clinpsy-032816-045244 28301765

[ref67] Miller, J.D. , Lynam, D.R. , McCain, J.L. , Few, L.R. , Crego, C. , Widiger, T.A. , & Campbell, W.K. (2016). Thinking structurally about narcissism: An examination of the five-factor narcissism inventory and its components. Journal of Personality Disorders, 30, 1–18. 10.1521/pedi_2015_29_177 25710734

[ref69] Miltner, W. , Lemke, U. , Weiss, T. , Holroyd, C. , Scheffers, M. , & Coles, M. (2003). Implementation of error-processing in the human anterior cingulate cortex: A source analysis of the magnetic equivalent of the error-related negativity. Biological Psychology, 64, 157–166. 10.1016/s0301-0511(03)00107-8 14602360

[ref68] Miltner, W. H. R. , Braun, C. H. , & Coles, M. G. H. (1997). Event-related brain potentials following incorrect feedback in a time-estimation task: Evidence for a “generic” neural system for error detection. Journal of Cognitive Neuroscience, 9, 788–798. 10.1162/jocn.1997.9.6.788 23964600

[ref70] Morf, C. C. , & Rhodewalt, F. (2001). Unraveling the paradoxes of narcissism: A dynamic self-regulatory processing model. Psychological Inquiry, 12, 177–196. 10.1207/s15327965pli1204_1

[ref71] Moser, J. S. , Moran, T. P. , Schroder, H. S. , Donnellan, M. B. , & Yeung, N. (2013). On the relationship between anxiety and error monitoring: A meta-analysis and conceptual framework. Frontiers in Human Neuroscience, 7, 466. 10.3389/fnhum.2013.00466 23966928 PMC3744033

[ref72] Mück, M. , Ohmann, K. , Dummel, S. , Mattes, A. , Thesing, U. , & Stahl, J. (2020). Face perception and narcissism: Variations of event-related potential components (P1 & N170) with admiration and rivalry. Cognitive, Affective, & Behavioral Neuroscience, 20, 1041–1055. 10.3758/s13415-020-00818-0 PMC7497513 32803683

[ref73] Murphy, P. R. , Robertson, I. H. , Allen, D. , Hester, R. , & O’Connell, R. G. (2012). An electrophysiological signal that precisely tracks the emergence of error awareness. Frontiers in Human Neuroscience, 6, 1–16. 10.3389/fnhum.2012.00065 22470332 PMC3314233

[ref74] Nieuwenhuis, S. , Holroyd, C. B. , Mol, N. , & Coles, M. G. (2004). Reinforcement-related brain potentials from medial frontal cortex: Origins and functional significance. Neuroscience and Biobehavioral Reviews, 28, 441–448. 10.1016/j.neubiorev.2004.05.003 15289008

[ref75] Nieuwenhuis, S. , Ridderinkhof, K. R. , Blom, J. , Band, G. P. , & Kok, A. (2001). Error-related brain potentials are differentially related to awareness of response errors: Evidence from an antisaccade task. Psychophysiology, 38, 752–760. 10.1111/1469-8986.3850752 11577898

[ref76] Overbeek, T. J.M. , Nieuwenhuis, S. , & Ridderinkhof, K. R. (2005). Dissociable components of error processing: On the functional significance of the Pe vis-à-vis the ERN/Ne. Journal of Psychophysiology, 19, 319–329. 10.1027/0269-8803.19.4.319

[ref77] Perrin, F. , Pernier, J. , Bertrand, O. , & Echalli, J. F. (1989). Spherical splines for scalp potential and current density mapping. Electroencephalography and Clinical Neurophysiology, 72, 184–187. 10.1016/0013-4694(89)90180-6 2464490

[ref78] Pincus, A. L. , Cain, N. M. , & Wright, A. G. (2014). Narcissistic grandiosity and narcissistic vulnerability in psychotherapy. Personality Disorders, 5, 439–443. 10.1037/per0000031 24446581

[ref79] Pinheiro, J. , Bates, D. , DebRoy, S. , Sarkar, S. , & R Development Core Team. (2010). Nlme: Linear and Nonlinear Mixed Effects Models. R package version (Version 3.1–97). Retrieved from https://CRAN.R-project.org/package=lme.

[ref80] Pontifex, M. B. , Scudder, M. R. , Brown, M. L. , O’Leary, K. C. , Wu, C. T. , Themanson, J. R. , & Hillman, C. H. (2010). On the number of trials necessary for stabilization of error-related brain activity across the life span. Psychophysiology, 47, 767–773. 10.1111/j.1469-8986.2010.00974.x 20230502

[ref81] Potts, G. F. (2011). Impact of reward and punishment motivation on behavior monitoring as indexed by the error-related negativity. International Journal of Psychophysiology, 81, 324–331. 10.1016/j.ijpsycho.2011.07.020 21855583 PMC3195890

[ref82] Pourtois, G. , Vocat, R. , N’diaye, K. , Spinelli, L. , Seeck, M. , & Vuilleumier, P. (2010). Errors recruit both cognitive and emotional monitoring systems: Simultaneous intracranial recordings in the dorsal anterior cingulate gyrus and amygdala combined with fMRI. Neuropsychologia, 48, 1144–1159. 10.1016/j.neuropsychologia.2009.12.020 20026086

[ref83] Rabbitt, P. (1968). Three kinds of error-signalling responses in a serial choice task. The Quarterly Journal of Experimental Psychology, 20, 179–188. 10.1080/14640746808400146 5653421

[ref84] Rabbitt, P. (2002). Consciousness is slower than you think. The Quarterly Journal of Experimental Psychology. A, Human Experimental Psychology, 55, 1081–1092. 10.1080/02724980244000080 12420985

[ref85] Raskin, R. , Novacek, J. , & Hogan, R. (1991). Narcissism, self-esteem, and defensive self-enhancement. Journal of Personality, 59, 19–38. 10.1111/j.1467-6494.1991.tb00766.x 2037962

[ref86] Rhodewalt, F. , & Morf, C. C. (1998). On self-aggrandizement and anger: A temporal analysis of narcissism and affective reactions to success and failure. Journal of Personality and Social Psychology, 74, 672–685. 10.1037//0022-3514.74.3.672 9523411

[ref87] Rhodewalt, F. , Tragakis, M. W. , & Finnerty, J. (2006). Narcissism and self-handicapping: Linking self-aggrandizement to behavior. Journal of Research in Personality, 40, 573–597. 10.1016/j.jrp.2005.05.001

[ref88] Ridderinkhof, K. R. , de Vlugt, Y. , Bramlage, A. , Spaan, M. , Elton, M. , Snel, J. , & Band, G. P. (2002). Alcohol consumption impairs detection of performance errors in mediofrontal cortex. Science, 298, 2209–2211. 10.1126/science.1076929 12424384

[ref89] Ridderinkhof, K. R. , Ullsperger, M. , Crone, E. A. , & Nieuwenhuis, S. (2004). The role of the medial frontal cortex in cognitive control. Science, 306, 443–447. 10.1126/science.1100301 15486290

[ref90] Sachse, R. (2013). Persönlichkeitsstörungen: Leitfaden für eine psychologische Psychothera-pie. Göttingen: Hogrefe, 2. Auflage.

[ref91] Saunders, B. , Milyavskaya, M. , & Inzlicht, M. (2015). What does cognitive control feel like? Effective and ineffective cognitive control is associated with divergent phenomenology. Psychophysiology, 52, 1205–1217. 10.1111/psyp.12454 26041054

[ref92] Scalabrini, A. , Huang, Z. , Mucci, C. , Perrucci, M. G. , Ferretti, A. , Fossati, A. , Romani, G. L. , Northoff, G. , & Ebisch, S. (2017). How spontaneous brain activity and narcissistic features shape social interaction. Scientific Reports, 7, 9986. 10.1038/s41598-017-10389-9 28855682 PMC5577167

[ref93] Sommer, K. L. , Kirkland, K. L. , Newman, S. R. , Estrella, P. , & Andreassi, J. L. (2009). Narcissism and cardiovascular reactivity to rejection imagery. Journal of Applied Social Psychology, 39, 1083–1115. 10.1111/j.1559-1816.2009.00473.x

[ref94] Stahl, J. , Acharki, M. , Kresimon, M. , Völler, F. , & Gibbons, H. (2015). Perfect error processing: Perfectionism-related variations in action monitoring and error processing mechanisms. International Journal of Psychophysiology, 97, 153–162. 10.1016/j.ijpsycho.2015.06.002 26071226

[ref95] Stahl, J. , Mattes, A. , Hundrieser, M. , Kummer, K. , Mück, M. , Niessen, E. , Porth, E. , Siswandari, Y. , Wolters, P. , & Dummel, S. (2020). Neural correlates of error detection during complex response selection: Introduction of a novel eight-alternative response task. Biological Psychology, 156, 107969. 10.1016/j.biopsycho.2020.107969 33058968

[ref96] Steinhauser, M. , & Yeung, N. (2010). Decision processes in human performance monitoring. The Journal of Neuroscience, 30, 15643–15653. 10.1523/JNEUROSCI.1899-10.2010 21084620 PMC3073548

[ref97] Steinhauser, M. , & Yeung, N. (2012). Error awareness as evidence accumulation: Effects of speed-accuracy trade-off on error signaling. Frontiers in Human Neuroscience, 6, 240. 10.3389/fnhum.2012.00240 22905027 PMC3417303

[ref98] Stucke, T. S. (2003). Who’s to blame? Narcissism and self-serving attributions following feedback. European Journal of Personality, 17, 465–478. 10.1002/per.497

[ref99] Trujillo, L. T. , & Allen, J. J. (2007). Theta EEG dynamics of the error-related negativity. Clinical neurophysiology, 118, 645–668. 10.1016/j.clinph.2006.11.009 17223380

[ref100] Twisk, J. W. R. (2006). Applied multilevel analysis: A practical guide. Cambridge: Cambridge University Press.

[ref109] Vidal, F. , Hasbroucq, T. , Grapperon, J. , & Bonnet, M. (2000). Is the ‘error negativity’ specific to errors? Biological Psychology, 51, 109–128. 10.1016/s0301-0511(99)00032-0 10686362

[ref101] Vocat, R. , Pourtois, G. , & Vuilleumier, P. (2008). Unavoidable errors: A spatio-temporal analysis of time-course and neural sources of evoked potentials associated with error processing in a speeded task. Neuropsychologia, 46, 2545–2555. 10.1016/j.neuropsychologia.2008.04.006 18533202

[ref102] Wegner, D. M. , & Zanakos, S. (1994). Chronic thought suppression. Journal of Personality, 62, 616–640. 10.1111/j.1467-6494.1994.tb00311.x 7861307

[ref103] Weinberg, A. , Dieterich, R. , & Riesel, A. (2015). Error-related brain activity in the age of RDoC: A review of the literature. International Journal of Psychophysiology, 98, 276–299. 10.1016/j.ijpsycho.2015.02.029 25746725

[ref104] Weinberg, A. , Riesel, A. , & Hajcak, G. (2012). Integrating multiple perspectives on error-related brain activity: The ERN as a neural indicator of trait defensive reactivity. Motivation and Emotion, 36, 84–100. 10.1007/s11031-011-9269-y

[ref105] Xiao, Z. , Wang, J. , Zhang, M. , Li, H. , Tang, Y. , Wang, Y. , Fan, Q. , & Fromson, J. A. (2011). Error-related negativity abnormalities in generalized anxiety disorder and obsessive-compulsive disorder. Progress in Neuro-Psychopharmacology & Biological Psychiatry, 35, 265–272. 10.1016/j.pnpbp.2010.11.022 21111024

[ref106] Yang, Z. , Sedikides, C. , Gu, R. , Luo, Y. , Wang, Y. , & Cai, H. (2018a). Narcissism and risky decisions: A neurophysiological approach. Social Cognitive and Affective Neuroscience, 13, 889–897. 10.1093/scan/nsy053 30016494 PMC6123519

[ref107] Yang, Z. , Sedikides, C. , Gu, R. , Luo, Y. L.L. , Wang, Y. , Yang, Y. , Wu, M. , & Cai, H. (2018b). Communal narcissism: Social decisions and neurophysiological reactions. Journal of Research in Personality, 76, 64–73. 10.1016/j.jrp.2018.07.003

[ref108] Zeigler-Hill, V. , Myers, E. M. , & Clark, C. B. (2010). Narcissism and self-esteem reactivity: The role of negative achievement events. Journal of Research in Personality, 44, 285–292. 10.1016/j.jrp.2010.02.005

