# Supplementary material for: Narcissism and the perception of failure – evidence from the error-related negativity and the error positivity
Source: Personal Neurosci. 2023 Feb 9;6:e2. doi: 10.1017/pen.2022.7 (PMC9947629; doi:10.1017/pen.2022.7)
Supplement: Supplementary file 1 [file S2513988622000074sup001.pdf]

Appendix

Multilevel model assessing the predictive value of Admiration and Rivalry on the P_e

	b	***SE b***	**95% CI**	***p***
Intercept	0.091	0.029	0.035, 0.147	0.002**
Number of Errors	-0.001	0.001	-0.002, 0.001	0.467
Session Type	0.025	0.025	-0.024, 0.074	0.326
Response Type	0.300	0.025	0.251, 0.349	< 0.001***
Admiration	-0.007	0.037	-0.080, 0.065	0.846
Rivalry	0.006	0.041	-0.075, 0.086	0.887
Admiration x Rivalry	-0.028	0.034	-0.094, 0.038	0.412
Sessions Type x Response Type	-0.016	0.036	-0.086, 0.053	0.650
Session Type x Admiration	0.002	0.032	-0.059, 0.064	0.940
Session Type x Rivalry	0.029	0.036	-0.040, 0.099	0.418
Response Type x Admiration	-0.025	0.032	-0.087, 0.036	0.429
Response Type x Rivalry	0.027	0.036	-0.042, 0.097	0.455
Response Type x Admiration x Rivalry	0.004	0.030	-0.053, 0.061	0.883
Session Type x Admiration x Rivalry	-0.018	0.030	-0.075, 0.038	0.533
Session Type x Response Type x Admiration	0.007	0.046	-0.081, 0.094	0.879
Session Type x Response Type x Rivalry	-0.004	0.051	-0.102, 0.095	0.945
Session Type x Response Type x Admiration x Rivalry	0.013	0.025	-0.068, 0.093	0.763

*$p < .05$, **$p < .01$, ***$p < .001$
